# Spontaneous Renal Hemorrhage in an Anticoagulated Patient With Prior Large-Vessel Vasculitis: A Wonder of Its Own

**DOI:** 10.7759/cureus.117326

**Published:** 2026-10-01

**Authors:** Diego de Silva, Achille Peiris, Amar Patel

**Affiliations:** 1 Nephrology, Balboa Nephrology Medical Group, San Diego, USA

**Keywords:** hypovolemic shock, spontaneous renal hematoma, spontaneous renal hemorrhage, vasculitis, wunderlich syndrome

## Abstract

Wunderlich syndrome (WS) is a spontaneous renal or perinephric hemorrhage occurring in the absence of trauma. Renal neoplasms are the most common cause, although vascular, infectious, cystic, and coagulopathic etiologies also occur. When active extravasation is detected on imaging, angiography with embolization is the preferred approach. We report a case of an 81-year-old woman with atrial fibrillation on apixaban and large-vessel vasculitis complicated by aortitis and a prior intramural aortic hematoma, who presented with four days of weakness, nausea, diarrhea, and right-sided abdominal pain, and was hypotensive at 71/56 mmHg. Contrast-enhanced computed tomography (CT) demonstrated a 12 × 12 cm right perinephric hematoma with active extravasation from a serpiginous vessel at the lower renal pole. Interventional radiology recommended medical management rather than embolization, citing the procedural risk associated with her aortitis. Treatment consisted of discontinuation of anticoagulation, reversal with prothrombin complex concentrate and vitamin K, transfusion of three units of packed red blood cells, crystalloid resuscitation, and vasopressor support. The course was complicated by oliguric acute kidney injury, with creatinine rising from 1.30 to 3.72 mg/dL, and by *Escherichia coli* bacteremia of urinary source. Serologic evaluation demonstrated a positive perinuclear anti-neutrophil cytoplasmic antibody (p-ANCA) result, with negative myeloperoxidase (MPO) and proteinase 3 (PR3) antibodies, and pulse methylprednisolone was administered. Urine output was reestablished with intravenous furosemide, vasopressors were discontinued by hospital day 4, and creatinine returned to 1.38 mg/dL before discharge on hospital day 7. This case demonstrates that conservative management can achieve hemodynamic and renal recovery in WS with active extravasation when vascular comorbidity precludes embolization.

## Introduction

Spontaneous renal or perinephric bleeding in the absence of trauma or iatrogenic hemorrhage from biopsy or surgical procedures is known as Wunderlich syndrome (WS) [1,2]. This represents a life-threatening condition that can rapidly cause hypovolemic shock and death [3]. The classic clinical picture, known as Lenk's triad, consists of sudden flank pain, a palpable flank mass, and signs of hypovolemic shock [4]. Renal tumors account for most cases of WS, with angiomyolipoma and renal cell carcinoma representing the leading benign and malignant causes, respectively [5]. Vascular abnormalities represent the second most frequent group of causes, with polyarteritis nodosa (PAN) accounting for the majority of these cases. Other less common etiologies include infection, coagulation disorders, and rupture of renal cysts, while a subset of cases remains idiopathic [2]. This report details a unique clinical encounter involving an 81-year-old female patient, notable for a background of anti-neutrophil cytoplasmic antibody (ANCA)-associated vasculitis (AAV) and ongoing apixaban therapy for atrial fibrillation, who suffered from sudden non-traumatic renal bleeding and underwent successful medical stabilization with anticoagulation discontinuation, circulatory support, and immunosuppression, without surgical intervention.

This case study aimed to underscore AAV as a rare but clinically significant vascular etiology of WS and to illustrate that carefully coordinated medical management, rather than surgical or angiographic intervention, can achieve favorable outcomes in anticoagulated patients presenting with this condition.

## Case presentation

An 81-year-old woman with a history of obesity, obstructive sleep apnea, gastroesophageal reflux disease, fibromyalgia, atrial fibrillation on apixaban, hypertension, hyperlipidemia, chronic lung disease requiring supplemental home oxygen, and giant cell arteritis with large-vessel involvement complicated by aortitis and a prior intramural aortic hematoma presented to the emergency department with four days of generalized weakness, nausea, diarrhea, and right upper-quadrant abdominal pain. She denied preceding trauma or recent surgical or invasive procedures.

On presentation, she was hypotensive, with a blood pressure of 71/56 mmHg, heart rate of 64 beats/min, and peripheral oxygen saturation of 93% while receiving supplemental oxygen at 4 L/min by nasal cannula. Abdominal examination was notable for right-sided tenderness. Initial laboratory evaluation demonstrated a hemoglobin concentration of 11.3 g/dL, leukocyte count of 18.1 × 10^3^/µL, serum creatinine of 1.30 mg/dL, blood urea nitrogen of 39 mg/dL, and venous lactate of 2.2 mmol/L. Contrast-enhanced computed tomography (CT) of the abdomen and pelvis demonstrated findings consistent with WS, including active contrast extravasation from a serpiginous vascular structure extending toward the lower pole of the right kidney and a 12 × 12 cm right perinephric hematoma producing significant renal compression (Figure 1).

**Figure 1:**
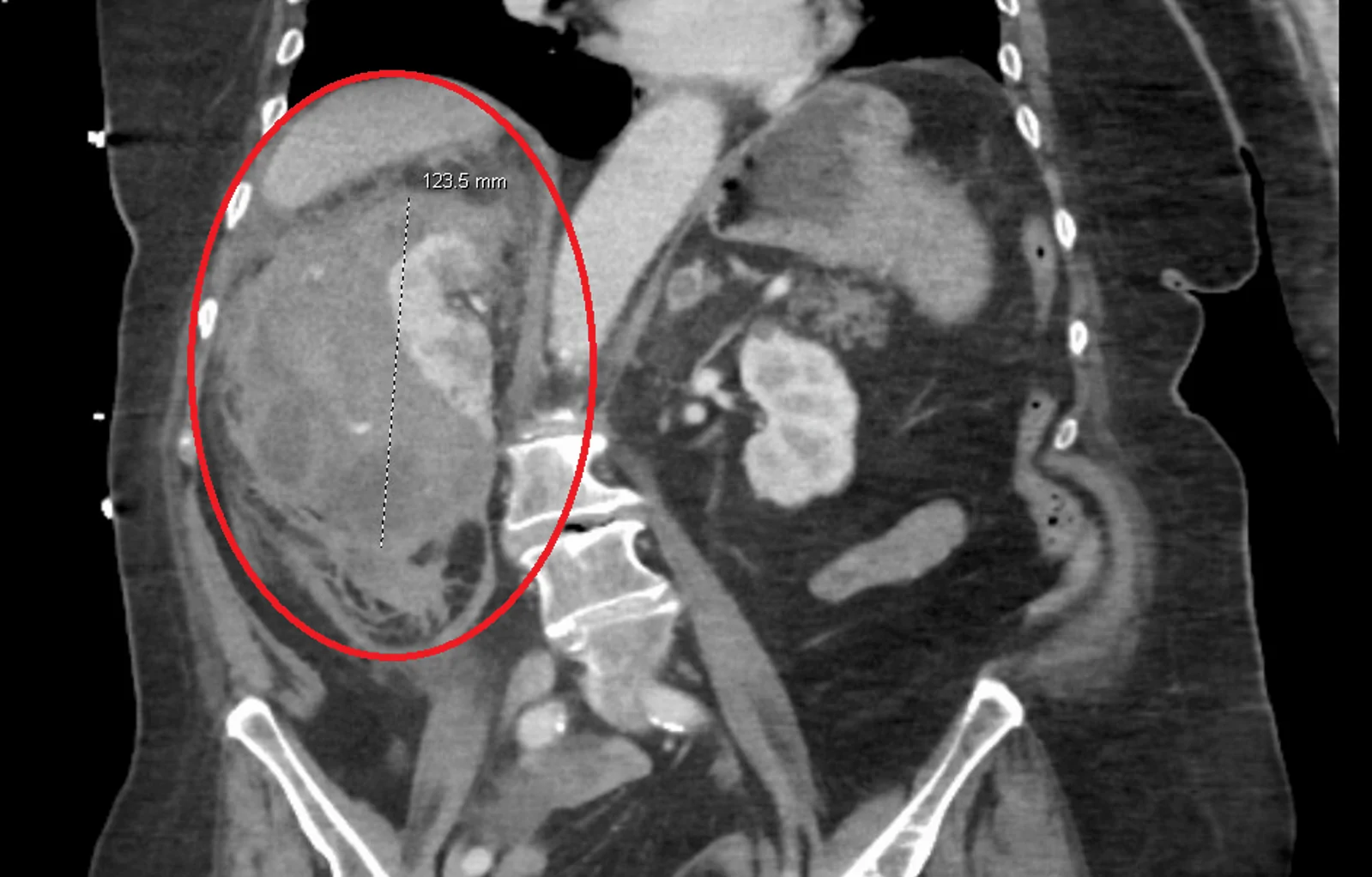
Abdominal CT scan on admission. The red circle indicates active bleeding/extravasation from the lower pole of the right kidney with a large right-sided perinephric hematoma compressing the right kidney. CT: computed tomography

Initial management included 1,000 mL of intravenous crystalloid, with improvement in blood pressure to 104/74 mmHg, as well as intravenous fentanyl for analgesia. Apixaban was discontinued, and four-factor prothrombin complex concentrate was administered for anticoagulation reversal. Vitamin K was also administered, although it was not considered a specific reversal agent for apixaban; factor Xa activity was noted to be slightly elevated. Interventional radiology was consulted on two occasions regarding renal angiography to further evaluate the possibility of a PAN-like vascular process. Angiography and embolization were deferred because advancing a catheter through a diseased aorta in the setting of prior aortitis was considered procedurally high risk, and the large right perinephric hematoma had distorted the renal vasculature to an extent that angiography was not expected to provide additional diagnostic information at that time. Repeat angiographic evaluation was considered reasonable after interval improvement of the hematoma. The patient was subsequently admitted to the intensive care unit on hospital day 0, where norepinephrine and vasopressin were initiated for circulatory support.

During the early hospitalization, her hemoglobin declined to 8.8 g/dL, and she developed oliguric acute kidney injury. Serum creatinine increased from 1.30 mg/dL on admission to 3.00 mg/dL on hospital day 1 and peaked at 3.72 mg/dL on hospital day 2. Conservative management remained the primary therapeutic strategy and consisted of discontinuation of anticoagulation, transfusion of a total of three units of packed red blood cells during the hospitalization, administration of prothrombin complex concentrate, balanced crystalloid resuscitation, and vasopressor support targeting a mean arterial pressure of at least 65 mmHg. Empiric antimicrobial therapy was also initiated. Operative intervention was not pursued because the patient stabilized with supportive treatment and did not demonstrate progressive hemodynamic deterioration requiring surgical management.

On hospital day 3, blood cultures grew *Escherichia coli*. Piperacillin-tazobactam was continued initially. The bacteremia was subsequently attributed to a urinary source after urine cultures yielded >100,000 CFU/mL of *Escherichia coli*, and antimicrobial therapy was narrowed to ceftriaxone in accordance with the susceptibility profile.

Rheumatology was consulted because of the patient’s history of large-vessel vasculitis with aortitis and concern for a possible renal vascular inflammatory process. Review of her clinical history and prior imaging suggested that the renal vascular abnormalities represented a new finding compared with previous CT studies. Given her history of aortitis and large-vessel vasculitis, interruption of corticosteroid therapy, and failure to initiate the previously planned steroid-sparing immunosuppressive treatment, intravenous methylprednisolone was initiated at 1 g daily for two days, followed by 80 mg intravenously daily and subsequently 30 mg intravenously twice daily. Rheumatologic evaluation demonstrated mild elevations in erythrocyte sedimentation rate (ESR) and C-reactive protein (CRP), along with elevated complement 3 (C3), complement 4 (C4), and total hemolytic complement (CH50) levels. Perinuclear ANCA (p-ANCA) was positive, whereas myeloperoxidase (MPO) and proteinase 3 (PR3) antibodies were negative. In the absence of clear evidence of glomerulonephritis and given the substantial procedural risk associated with the large renal hemorrhage, kidney biopsy and angiography were deferred. Thus, characterization was not possible at this time. 

The patient remained oliguric, with urine output decreasing to approximately 150 mL/day over a two-day period. Because there was no emergent indication for renal replacement therapy, nephrology recommended a trial of intravenous furosemide at a dose of 100 mg. She demonstrated a favorable diuretic response, producing approximately 1,800 mL of urine overnight. Scheduled intravenous furosemide at 80 mg twice daily was subsequently continued to maintain adequate urine output. Vasopressor support was discontinued by hospital day 4.

Hemoglobin remained relatively stable during the remainder of the hospitalization without evidence of recurrent clinically significant hemorrhage (Table 1). Renal function progressively improved, with serum creatinine decreasing to 3.10 mg/dL on hospital day 4, 2.30 mg/dL on day 5, 1.59 mg/dL on day 6, and 1.38 mg/dL on day 7 (Table 2).

**Table 1:** Serial hematologic laboratory findings during hospitalization. * indicates abnormal values. WBC: white blood cell count; PLT: platelets; HGB: hemoglobin

Laboratory parameter	Units	Day 4 03:52	Day 4 17:38	Day 5 04:06	Day 5 18:14	Day 6 06:15	Day 6 17:57	Day 7 04:42	Day 7 09:13
WBC	K/mcL	12.7*	9.8	10.1	13.8*	16.1*	20.2*	21.8*	-
HGB	g/dL	7.6*	7.6*	7.8*	9.7*	9.7*	9.8*	10.7*	10.3*
Hematocrit	%	22.0*	22.4*	23.0*	29.5*	29.1*	29.2*	32.3*	-
PLT	K/mcL	200	204	211	248	268	275	290	-

**Table 2:** Serial serum chemistry findings during hospitalization. * indicates abnormal values. BUN: blood urea nitrogen; CO_2_: carbon dioxide

Laboratory parameter	Units	Day 1 14:01	Day 2 04:56	Day 3 06:19	Day 3 18:15	Day 4 03:52	Day 4 13:08	Day 5 04:06	Day 6 06:15	Day 7 04:42	Day 7 09:13
Sodium	mmol/L	140	142	143	-	142	-	143	137	134*	-
Sodium, whole blood	mmol/L	-	-	-	-	-	-	-	-	-	133*
Potassium	mmol/L	5.2*	4.6	3.6	4.2	3.4*	3.4*	4.1	3.5	3.7	-
Potassium, whole blood	mmol/L	-	-	-	-	-	-	-	-	-	3.1*
Chloride	mmol/L	108	110	106	-	103	-	103	96*	94*	-
Chloride, whole blood	mmol/L	-	-	-	-	-	-	-	-	-	94*
CO_2_	mmol/L	16*	16*	21*	-	25	-	26	28	29	-
BUN	mg/dL	49*	60*	69*	-	69*	-	71*	68*	64*	-
Creatinine	mg/dL	3.00*	3.72*	3.70*	-	3.10*	-	2.30*	1.59*	1.38*	-
Calcium	mg/dL	8.2*	8.0*	7.6*	-	7.8*	-	8.1*	8.9	8.8	-
Glucose	mg/dL	158*	173*	159*	-	157*	-	148*	137*	130*	-

On hospital day 7, the patient remained rate-controlled on metoprolol 50 mg daily and was discharged without resumption of anticoagulation. She was transitioned to oral prednisone. Given the recent life-threatening renal hemorrhage, anticoagulation was considered contraindicated at discharge because the risk of recurrent bleeding was judged to outweigh the immediate thromboembolic benefit. Reassessment of anticoagulation was planned after interval imaging demonstrated improvement or resolution of the hematoma. Catheter-based renal angiography was recommended when clinically feasible to further evaluate for an underlying renal vascular abnormality or vasculitic process. Long-term steroid-sparing immunosuppressive therapy was also recommended, with selection of the specific agent to depend on subsequent vascular imaging and whether findings suggestive of a PAN-like medium-vessel vasculitis were identified.

## Discussion

Spontaneous renal hemorrhage, also referred to as WS, is an uncommon but potentially fatal condition for which management strategies vary considerably according to the clinical scenario and underlying cause. WS can arise from a broad range of renal disorders, both neoplastic and non-neoplastic. Renal tumors are the leading cause, with angiomyolipoma most often identified among benign lesions and renal cell carcinoma among malignant lesions. Additional etiologies include vascular abnormalities such as PAN, renal artery aneurysms, arteriovenous malformations or fistulas, and venous thrombosis, as well as cystic kidney disease, infection, nephrolithiasis, nephritis, and coagulation disorders [6]. 

Published experience suggests that selected patients can be managed nonoperatively with analgesia and close clinical surveillance [7]. However, because spontaneous renal bleeding can progress rapidly and become life-threatening, escalation to more invasive treatment may be required when hemorrhage persists or clinical instability develops [7]. When imaging demonstrates ongoing contrast extravasation, catheter angiography with selective embolization is generally favored, while partial or radical nephrectomy is typically reserved for cases involving an underlying tumor or bleeding that cannot be controlled by less invasive measures [8]. 

In our case, the patient presented with hypovolemic shock, right flank pain, anemia, acute kidney failure, and active extravasation from serpiginous vessels on the lower pole of the kidney. Based on clinical presentation and upon imaging confirmation, aggressive intervention could have been warranted. Compared with nephrectomy, selective renal artery embolization offers a less invasive means of controlling hemorrhage and may better preserve renal function than conservative management alone [9]. However, the presence of vasculitis represented an additional layer of clinical complexity, rendering selective embolization of the renal arteries risky and beyond achievable grounds. Management of spontaneous renal hemorrhage should be tailored to its underlying cause. When AAV is implicated, treatment may rely primarily on medical therapy with glucocorticoids and other immunosuppressive agents, together with supportive measures such as volume resuscitation and transfusion as needed. The intensity of immunosuppression should reflect the overall degree of vasculitic activity, and a less aggressive regimen may be appropriate when there is limited evidence of active systemic disease [10].

This patient experienced improvement in abdominal pain, stabilization of vital signs, and cessation of hemoglobin decline, with subsequent improvement on pulse glucocorticoids. Nephrectomy and embolization were ultimately not pursued because hemodynamic stability was achieved with conservative management and there was no evidence of ongoing clinically significant hemorrhage.

## Conclusions

WS is an uncommon but potentially fatal clinical emergency in which early diagnosis and management tailored to the individual patient are essential. Although active extravasation often prompts angiographic embolization, this case demonstrates that carefully selected patients may achieve favorable outcomes with conservative treatment when procedural risk is considered prohibitive. Despite presenting with hemorrhagic shock, a large compressive perinephric hematoma, and subsequent oliguric acute kidney injury, our patient recovered with anticoagulation reversal, transfusion, hemodynamic support, corticosteroid therapy, and close multidisciplinary monitoring. Her renal function improved substantially without embolization, nephrectomy, or renal replacement therapy. The coexistence of anticoagulation and prior large-vessel vasculitis further underscores the importance of considering multiple potential etiologies and tailoring intervention to the individual patient’s clinical stability, vascular risk, and underlying comorbidities. Long-term vascular imaging remains essential to clarify the underlying cause and guide future immunosuppressive therapy.
